# The Efficiency of Spa Rehabilitation in Chronic Ischemic Stroke Patients—Preliminary Reports

**DOI:** 10.3390/brainsci11040501

**Published:** 2021-04-15

**Authors:** Bogumiła Pniak, Justyna Leszczak, Jadwiga Kurczab, Aleksandra Krzemińska, Joanna Pięta, Agnieszka Plis, Ewelina Czenczek-Lewandowska, Agnieszka Guzik

**Affiliations:** 1 Excelsior Health and Rehabilitation Hospital, Al. Torosiewicza 2, 38-440 Iwonicz-Zdrój, Poland; gabipniak@vp.pl (B.P.); milak_123@wp.pl (J.K.); okrzemien159@gmail.com (A.K.); joannapieta1@gmail.com (J.P.); a.plis8833@gmail.com (A.P.); 2 Institute of Health Sciences, Medical College, University of Rzeszów, ul. Kopisto 2a, 35-959 Rzeszów, Poland; e.czenczek@univ.rzeszow.pl (E.C.-L.); agnieszkadepa2@wp.pl (A.G.)

**Keywords:** quality of life, stroke, spa rehabilitation, functional efficiency, spa hospital

## Abstract

Background: Rehabilitation-oriented therapy after a stroke must continue in various forms as a life-long effort. Aim: The study investigated the impact of spa rehabilitation on the quality of life and functional efficiency in patients after an ischemic stroke at a chronic stage of recovery. Methods: The assessment was carried out in a spa resort in southeastern Poland. It involved 32 patients with strokes who participated in a three-week rehabilitation program. Three examinations were performed: upon admission, on the day of discharge and at a two-month follow-up. The quality of life and functional efficiency were assessed with the WHOQOL-BREF and Barthel Index. Results: The quality of life was significantly higher in Exam II compared with Exam I (*p* < 0.001), and improvement was retained at the follow-up. The Barthel scores were higher in Exam II compared with Exam I (79.84 vs. 68.59), while the differences between the scores in Exams II and III were small (*p* = 0.039). Conclusions: Three-week spa rehabilitation seems to favorably affect the functional efficiency and quality of life after a stroke. The effects appear to be long-term. The gender, age and time from stroke onset do not seem to impact short-term effects. However, long-term effects are related to the time from stroke onset.

## 1. Introduction

Rehabilitation-oriented therapy after a stroke should be initiated as soon as possible and continue as a life-long effort, because in most individuals, a stroke leads to disability. However, one should bear in mind that during the first few weeks following a stroke, rehabilitation should not be too intensive [1]. The purpose of physiotherapy applied post-stroke is to improve the patient’s functional efficiency and restore their self-sufficiency and psychophysical balance as much as possible [2,3].

Specialist rehabilitation centers, referred to as “spa facilities”, located in areas with favorable environmental conditions and natural therapeutic resources, provide excellent conditions for recovery. Such facilities may continue comprehensive treatments Please carefully check the accuracy of names and affiliations. Changes will not be possible after proofreading. specially designed for patients who have lost their functional capacities [4,5]. Furthermore, those participating in “retreat-based therapy” have an opportunity to relax, experience a new environment and establish new social contacts, all of which may promote their recovery in various domains [6]. Spa rehabilitation programs include a wide range of balneotherapies, making use of natural mineral waters and administered at various temperatures. Other treatments apply peloids or gases with medicinal properties. Hydrogen sulphides, carbonic acid, as well as iodine and bromine baths improve microcirculation, stimulate the metabolism and alleviate many adverse symptoms, including spasticity. Treatments based on balneotherapy have been found to strengthen immunity and support faster recovery [7] as well as normalize blood pressure. Carbonic acid and hydrogen sulphide baths are helpful for reducing systolic blood pressure [8]. Physical exercise performed in a rehabilitation pool has been found to decrease pain and reduce spasticity [9,10]. In addition to balneotherapy, spa rehabilitation programs include a wide range of treatments based on a variety of specialized physiotherapeutic methods and kinesitherapy (e.g., proprioceptive neuromuscular facilitation, neurodevelopmental treatment [11] and constraint-induced movement therapy), as well as tools applying biofeedback. Biofeedback-supported devices have been shown to promote the recovery of lost functions in patients with neurological problems (e.g., manifested by gait impairment) [12,13]. Beneficial effects have also been confirmed in the case of complementary physical treatments, such as tonolysis, low-frequency variable magnetic fields, functional electrical stimulation (FES) and cryotherapy [14].

According to the definition from the World Health Organization (WHO), quality of life is an “individual’s perception of their position in life in the context of the culture and value systems in which they live and in relation to their goals, expectations, standards and concerns”. The related indicators include one’s ability to play life roles, as well as one’s adaptability, good mental condition and functioning in society [2,15]. Therapies designed for people presenting post-stroke dysfunctions are primarily aimed at improving the musculoskeletal system. However, efforts to improve the patient’s functioning in the psychological and social domains are equally necessary to enable optimum recovery after a stroke incident [16]. Quality of life after a stroke, as well as the effects of post-stroke rehabilitation, have been extensively investigated. Indeed, the focus on neurosciences and various types of post-stroke rehabilitation increased significantly during the so-called Decade of the Brain. Advancements in neurobiology related to the complexity of effects resulting from central nervous system damage, as well as brain plasticity, provided better insight into the mechanisms of regeneration and functional recovery in patients after strokes [17,18,19]. It has been established that the largest progress in the recovery of functional capacities can be expected during the early period after a stroke, when patients frequently present considerable improvements in neuromotor functions [20,21] due to the fact that recovery-related changes within the ischemic penumbra adjacent to the focal lesion occur relatively rapidly following onset, and later, the process slows down [22,23]. Given the above, the present study was designed to focus on a population of patients at a chronic stage of recovery (i.e., over six months from stroke onset). At this stage, patients tend to present persistent patterns and are largely accustomed to functioning in their own environment. Despite this, the authors hypothesized that significant improvement in quality of life and functional efficiency may still be feasible at this stage of recovery and that changes in these areas would be promoted by spa rehabilitation. The above hypothesis is encouraged by research findings showing that interneuronal connections are constantly remodeled by physical activity. If complex motor activities are performed systematically, the area of cortical representation of such activity increases. This suggests that brain plasticity may be enhanced as a result of various types of training [24,25]. This process has already been investigated with regard to hospital and ambulatory post-stroke rehabilitation [26,27,28,29,30], but not in relation to spa rehabilitation.

In summary, a review of the literature confirms that the entire post-stroke treatment process, including rehabilitation, is primarily conducted in hospitals, clinics and outpatient facilities. However, there are no studies assessing the effectiveness of treatments continued in spa facilities, which can create favorable conditions for the recovery of full physical and mental efficiency [2].

In view of the above, the aim of the current study was to assess the effects of a spa rehabilitation program, reflected in the quality of life and functional efficiency in patients after ischemic strokes at a chronic stage of recovery. The second aim of the study was to assess the effect of spa rehabilitation relative to the age, gender and time from stroke onset.

## 2. Materials and Methods

The study was conducted in accordance with the ethical rules of the Helsinki Declaration, and it was approved by the local bioethics commission (consent no. 2015/10/03). Written consent was obtained from all participants in the study.

### 2.1. Study Group

The study group was selected from a database of a spa facility in southeastern Poland.

The inclusion criteria were as follows: age over 48 years, completed first ischemic stroke, completion of a three-week spa rehabilitation program and patient’s consent to participate in the study. The study included patients whose functional performance on admission was reflected by a score of 85 on the Barthel Scale, a score of 2 or 3 according to the Rankin Scale, and a score of 3 or 4 according to the Functional Ambulation Category (FAC). None of the patients enrolled for the study had previously participated in health resort-based therapy programs.

The following exclusion criteria were applied: hemorrhagic stroke, significant random events during observation (such as the death of a family member or divorce), diagnosis of other diseases that may affect the quality of life (such as an unstable medical condition, orthopedic or rheumatic disorders, oncological and other neurological diseases (e.g., Parkinson’s disease or multiple sclerosis), pain and inflammation in the musculoskeletal system and cognitive impairment), other forms of therapy implemented during the observation period and refusal to participate in Exam II or III.

All of the patients included in the study participated in a 3 week health resort-based rehabilitation program. The patients were examined three times by the therapeutic team. The first examination took place on the first day of therapy (Exam I), and the second (Exam II) was conducted at the end of the 3 week rehabilitation program on the day of discharge from the spa facility. The third examination (Exam III, the follow-up) was performed two months after discharge from the spa facility.

### 2.2. Rehabilitation Program

The spa rehabilitation program was continued for three weeks from Monday to Friday (a total of 15 days of treatment). Each patient staying in the spa facility participated in a comprehensive rehabilitation program conducted during the day, lasting from 120 to 150 min. The rehabilitation program was individually selected by the therapeutic team for the needs of each patient. All patients participated in morning training, individual exercises, group gymnastics in water, mud therapy, hydrotherapy with mineral water and crenotherapy (hydrogen sulphide and inorganic sulphide water, hydrogen chloride-bicarbonate-sodium, iodide and acidified water). The specifications of the hydrogen sulphide and inorganic sulphide water were Na^+^, K^+^, Li^+^, Ca^2+^, Mg^2+^, Fe^2+^, M^−^, Cl^−^. The mineralization of this water was 710–820 mg/dm^3^. The level of hydrogen sulphide was 34.7–49.6 mg/dm^3^. The specifications of the hydrogen chloride, sodium carbonate, iodide and acidified water were Na^+^, K^+^, Li^+^, Ca^2+^, Mg^2+^, Fe^2+^, Sr^2+^, Ba^2+^, M^−^, Cl^−^, Br^−^, J^−^. The mineralization of the water was 10,806.3974 mg/dm^3^.

### 2.3. Assessment Tools

The Barthel Index was used to assess the patients’ functional performance. The scale allows the assessment of 10 basic skills related to activities of daily living (feeding, transfers (bed to chair and back), personal hygiene, toilet use, bathing, mobility on level surfaces, stair climbing, dressing and undressing, bladder control and bowel control). The patient may receive 0, 5 or 10 points for each activity. The index enables the assessment of performance on a 100 point scale, with lower scores corresponding to a patient’s poorer functional efficiency.

The WHOQOL-BREF questionnaire was applied to assess the patients’ quality of life. This tool makes it possible to determine the quality of life profile for the last 14 days in four domains: somatic, psychological, social and environmental. The answers to the questions included in the WHOQOL-BREF scale are scored on a five-point scale, with a higher number of points reflecting a better self-reported quality of life in a given area. The score obtained is converted into a 100 point scale, with a higher result deemed to reflect a higher quality of life [31].

### 2.4. Statistical Analysis

The sample size calculation procedure performed before the study took into account the number of patients with strokes staying at the spa facility per year. A fraction size of 0.9, a maximum error of 10% as well as a 95% confidence interval were adopted; as a result, a sample size of 30 subjects was determined. Thirty-two patients were included in the final analysis (31 in Exam III).

At the first stage, distributions of quantitative variables were assessed for normality using the Shapiro–Wilk test. Since satisfactory results were obtained, comparative analyses of the mean values were carried out using parametric tests, namely an independent sample *t*-test and a dependent sample *t*-test. Additionally, homogeneity of variances was examined using Levene’s test, and *p*-value adjustment was introduced if the assumption of homogeneity was not verified. McNemar’s test was applied to compare qualitative variables. The analyses were carried out for the whole group of patients, as well as relative to the gender, age and time from stroke onset. Relationships at *p* < 0.05 were considered statistically significant.

## 3. Results

### Flow of Participants

**Figure 1:**
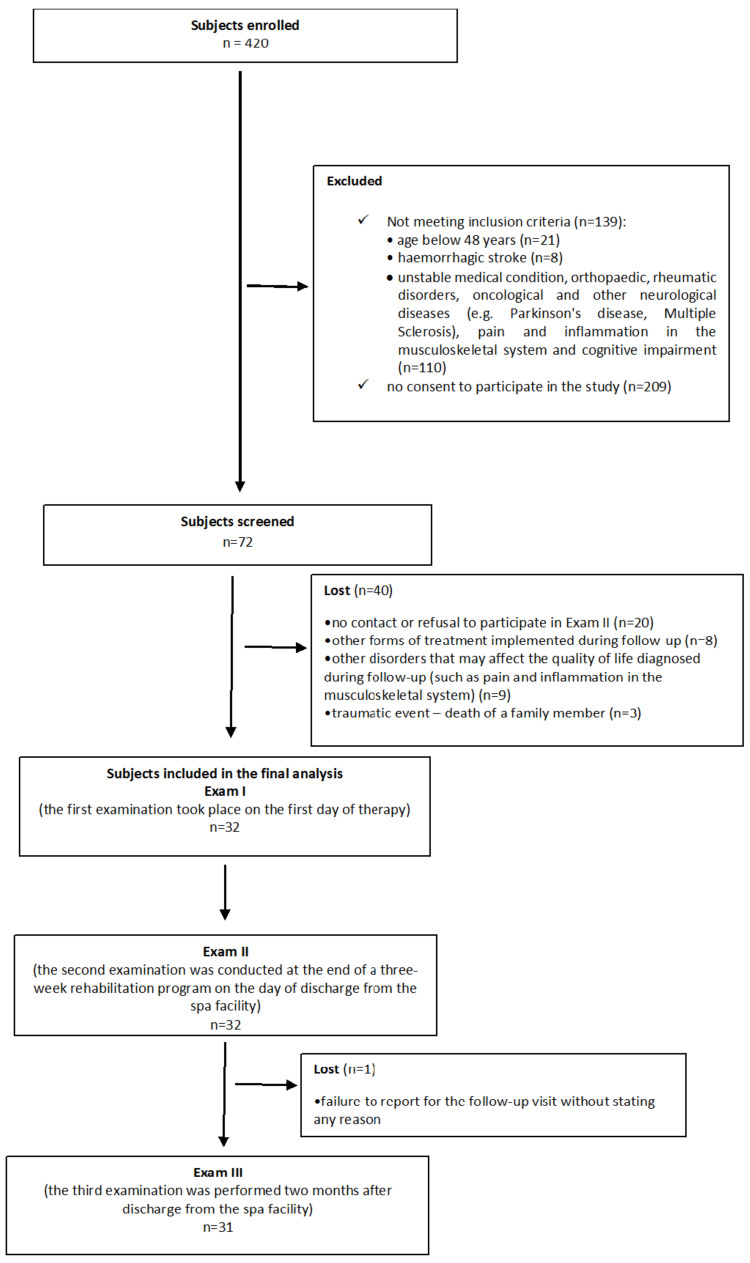
Flow diagram of the study population.

A total of 420 patients after strokes were staying at the spa facility during the studied period. The inclusion criteria were not met by 139 individuals, while 209 patients did not agree to participate in the study. Seventy-two subjects were examined, and subsequently, 40 individuals had to be discarded in the analyses (refusal to participate in the second exam; other forms of treatment implemented during the period preceding the follow-up exam; other disorders potentially affecting the quality of life identified during the follow-up, such as pain and inflammation in the musculoskeletal system; or a traumatic event, such as the death of family member). A total of 32 patients with ischemic strokes participated in both Exam I and Exam II, while 31 patients reported for Exam III (the follow-up). One person did not participate in the follow-up exam without stating any reason. The flow of the subjects through the study is shown in Figure 1.

A group of 16 women and 16 men aged 48–85 was included in the study. The mean age in the group was 66.31 years. The majority of the study participants were residents of urban areas (59.40%) and lived with their spouses and children (56.30), while 34.40% had higher educations (Table 1).

The quality of life in the somatic domain in Exam I on average amounted to 47.43 points and was significantly lower (*p* < 0.001) compared with Exam II (57.37 points) and slightly lower than in Exam III (49.74 points; *p* = 0.035). The difference between Exam II and Exam III was significant (*p* < 0.001). In the follow-up, the quality of life scores had not decreased to the level identified as the baseline, as the result of Exam III (49.74) was significantly higher (*p* = 0.035) than the related score in Exam I (47.43 points). Similar differences were observed in the remaining domains of life, including an increase in the quality of life in Exam II compared with Exam I and a significant decrease between Exam II and Exam III.

Regarding the Barthel Index, the findings showed a higher score in Exam II than Exam I (79.84 vs. 68.59), as well as a slight decrease in the score in Exam III (77.68) compared with Exam II (Table 2).

The short-term effects of the rehabilitation program (Effect I, the difference between Exam I and Exam II) were positive in all four domains of life. Likewise, the long-term outcomes (Effect II, the difference between Exam I and Exam III) were positive. However, the change in the quality of life (Effect III, the difference between the scores in Exam II and Exam III) was negative. The differences between the three specific effects of the rehabilitation program regarding quality of life were statistically significant (*p* < 0.05).

Regarding the Barthel Index, the largest effect of the rehabilitation program was shown by the difference between the scores in Exam I and Exam II (11.25 points). A lesser effect of the program was shown by the differences between Exam I and III (8.04 points), while the differences in Barthel scores between the exam at the end of the program and at the follow-up were reflected by the negative value of Effect III (−1.79 points).

Effect I and Effect II were not related to sex, age or time from stroke onset. Conversely, Effect III was related to the time from stroke onset in the somatic and psychological domains. A greater decrease in quality of life between Exams II and III was observed in patients 1–5 years from their strokes in the somatic (−9.67) and psychological domains (−8.58), compared with patients 5–10 years from stroke onset (−5.94 in somatic and −3.56 in the psychological domain).

Similarly, Effect I, reflected by the Barthel Index, was not related to sex, age or time from stroke onset. Effect II, in the case of the Barthel scores, was different for patients below 65 years of age (11.67) and those 65 and over (5.31); better improvement in the period between Exam I and Exam II was achieved by patients below 65 years of age (*p* = 0.007). Likewise, Effect II was higher (11.25) in patients 1–5 years from stroke onset, compared with those 5–10 years from stroke onset (5.63) (*p* = 0.032).

Effect III (i.e., the difference between Exam II and Exam III), in the case of Barthel scores, was reflected by a negative value (the results of the program were more likely to deteriorate) in patients aged 65 or more (−3.13) and in those 5–10 years after stroke onset (−3.13) (Table 3, Table S1).

## 4. Discussion

The aim of the study was to assess the impact of a therapy program applied in a health resort setting on the quality of life and functional efficiency of patients after chronic strokes. The second purpose of the study was to investigate the effect of spa rehabilitation relative to age, gender and time from a stroke. The current findings suggest that the quality of life and functional efficiency may be improved in patients at a chronic stage of recovery post-stroke if they receive spa rehabilitation. This is an important observation in view of the predominant evidence suggesting that significant improvements are mainly achieved by patients with strokes at early stages of recovery. Hence, it appears that comprehensive therapies administered in health resort settings may effectively complement conventional rehabilitation programs [32,33,34,35,36,37]. However, no studies have been published so far regarding the effectiveness of spa rehabilitation. This article presents the first scientific findings related to this matter.

In the current study, the patients’ quality of life was assessed using the WHOQOL-BREF scale. On the first day of their stay at the spa facility, the quality of life reported by the patients was lower than on the last day. The improvement achieved during the three-week program was sustained for two months, as shown by the follow-up examination. Although this is a subjective type of examination, its results provide important feedback to the therapist, as they reflect the patients’ satisfaction and self-perceived progress. What is more, improvement of the results was demonstrated in all the domains of functioning (i.e., somatic, psychological, social and environmental). A study by Shyu et al. showed that an improved quality of life was retained for three months after discharge from a hospital [38]. Similar trends were also reported by Lewthwaite et al. [39]. Likewise, Hopmann et al. noticed that as a result of hospital rehabilitation, the quality of life of patients improved, which is consistent with the current findings [40].

According to Bushnell, the risk of a stroke in young women is low; however, with age, both the incidence of and mortality associated with strokes gradually increase in the female population [41]. Because of this, it is important to assess sex-related differences in treatment outcomes. It has also been suggested that the patient’s age and gender should be taken into account when planning a rehabilitation program. Furthermore, according to some researchers, women who have suffered a stroke need longer hospitalization [42] and are more likely to develop symptoms of depression [43] compared with men. Furthermore, Phan et al. [44] and Carod-Artal showed that women reported a lower quality of life than men after a rehabilitation program [45]. The current study, however, did not show sex-related differences. Following the health resort-based treatment program, both men and women reported an improvement in quality of life. Likewise, according to Zawadzka et al. and Bolach et al., gender did not play a significant role in the quality of life after a stroke [46,47].

The impact of the patient’s age and time from stroke onset appears to be less controversial. The highest improvement in functioning generally is achieved by patients 3–6 months after the stroke incident [48]. The current findings show that patients who had experienced a stroke up to 5 years earlier reported higher improvement in their physical and mental health. As for age-related effects, a greater improvement in self-reliance in activities of daily living was achieved by younger people (i.e., under 65 years of age). Bejer et al. used the SS-QOL to assess the quality of life and reported that improvement after a rehabilitation program was observed in both younger and older patients [49], which is consistent with the present findings showing that the quality of life improved in both age groups.

The study shows that in terms of the quality of life, differences were observed in all the domains, with an increase in the quality of life in Exam II compared with Exam I and then a significant decrease between Exam II and Exam III. Regarding the Barthel Index, the findings showed a higher score in Exam II than Exam I, as well as a decrease in the score in Exam III compared with Exam II. However, the differences between Exams II and III were small, possibly reflecting the long-term effectiveness of the rehabilitation program. This may be associated with the fact that the environment experienced by patients in spa centers in Poland is more similar to a home setting than to a hospital setting. Home-style accommodations are provided to spa patients rather than hospital-type rooms. As a result, during their stay at the spa facility, the patients had an opportunity to improve their skills related to activities of daily living and to learn new self-care strategies. It is possible that which was stimulated in the spa setting was then brought to light in the activities of daily living at home. Perhaps the process leading to an improved quality of life requires more time, and the period preceding the follow-up exam made it possible for the skills acquired during the three weeks at the spa facility to be transposed into the home setting. This may explain the fact that improvements in Barthel Index are more robust.

Notably, the present study investigates the impact of a spa rehabilitation program on the quality of life and functional efficiency in patients at a chronic stage post-stroke. It has been established by numerous studies that the greatest progress in the recovery of functional abilities can be expected during the early period after a stroke, when patients frequently present considerable improvements in neuromotor functions. However, the process slows down later [20,21,22,23]. This may partly result from the adaptation of patients to functioning in their own environment. Therefore, we suspect that the impact of health resort-based rehabilitation programs on the quality of life and functional efficiency identified in the case of patients at a chronic stage of recovery may be lower than the effects that could be observed during the acute phase post-stroke. Hence, further research is needed to investigate this issue in patients at earlier stages after a stroke (e.g., 3–6 months from the incident), when therapy is generally the most beneficial. It would also be advisable to apply spa rehabilitation at acute phases post-stroke. At this point, spa rehabilitation programs in Poland are offered mainly to individuals at a chronic stage post-stroke, while patients up to 6 months from the onset of stroke symptoms generally receive conventional therapy [50,51,52,53,54,55,56]. In view of the current findings, which show considerable improvement in the quality of life and functional efficiency in patients at a chronic stage of recovery post-stroke, it can be expected that spa rehabilitation programs would even more significantly contribute to the recovery of patients at early stages after strokes. We also believe that although our findings showed a relatively minor difference between Exam III and Exam I in the quality of life, the change is clinically relevant because during Exam III, the patients reported improvement in the quality of life compared with that perceived by them at the time of their admission to the spa facility in Exam I. As a result, it can be assumed that the effect of the rehabilitation program was sustained for a longer period of time and was clinically relevant.

### Limitations and the Source of Potential Bias

The small sample size is the first limitation of this study. This is linked with a few factors. First of all, in accordance with the exclusion criteria, the study disregarded patients with such comorbidities as orthopedic or rheumatic disorders, oncological and other neurological diseases (e.g., Parkinson’s disease and multiple sclerosis), pain and inflammation in the musculoskeletal system and cognitive impairment. This criterion was applied due to the fact that there are many research reports showing that these medical conditions adversely affect the quality of life [57,58,59,60,61,62,63,64,65,66,67,68]. The authors wanted to recruit a homogenous group of subjects after strokes and with none of the above comorbidities in order to address the issue defined in the purpose of the study. This explains the large number of excluded patients. Another source of potential bias in our study may be related to the age criterion. Patients over 48 years of age were referred to our spa facility far more frequently than those below 48 years of age. This is linked with the fact that in Poland, this type of “retreat-based therapy” is mainly appreciated by and popular among older patients with various chronic conditions. During their stay at a spa facility, they can benefit from the specific local climate and enjoy various recreational options and leisure time activities [69] (e.g., dancing parties where they can meet other people). Because of this specificity of health resorts, the average age of the patients is rather high, and young people are a rarity there. Since those participating in health resort-based therapy in Poland predominantly include older people with chronic conditions [70], this option is far less often chosen by young people, who prefer rehabilitation programs offered by hospitals or outpatient clinics. Notably, however, the patient’s age was found to be extremely important in the outcomes, as reflected by the differences in the results observed in the patients below 65 years of age and those 65 and over. Given this, it is necessary to continue the research and assess the effectiveness of spa rehabilitation in younger patients with strokes (below 48 years of age). Thirdly, the presented results should be treated as preliminary and should be followed by a study involving a larger group. However, despite the small number of participants in this study, the sample size calculation that was carried out showed that our sample of participants was adequately powered for the feasibility study.

The next limitation of this study is the lack of a control group, which would have enabled a comparative analysis. This limitation is linked with the fact that all patients at this spa facility received therapy, which meant we could not select a control group of subjects receiving no treatments. However, in order to partly compensate for this limitation, we performed the third examination (as a follow-up) two months after discharge from the spa facility, which enabled assessment of the long-term effects of spa rehabilitation. Another limitation lies in the fact that the study did not assess the specific physiotherapeutic procedures administered to the patients at the spa facility for their effectiveness, reflected in the patients’ quality of life and functional efficiency, and there is a great need to conduct tests in other spas to verify the preliminary results obtained and ensure generalizability of the findings.

It is necessary to continue research into the effectiveness of spa rehabilitation for patients after strokes, reflected by changes in the quality of life and functional efficiency relative to the duration, type and intensity of therapy, which would involve a control group and a larger population. This would enable a more accurate and precise assessment of the impact of such factors as the gender, age or time from the stroke. Similar tests should be carried out in other health resorts to verify these preliminary results. It would also be interesting to compare the effects of health resort-based therapy and outpatient treatment programs applied to patients at a similar stage of recovery after a stroke.

## 5. Conclusions

The three-week rehabilitation program in a health resort setting led to a significant improvement in the functional efficiency and quality of life after a stroke. The obtained effect appears to be long-term, as it was sustained in the follow-up examination. If these findings are confirmed in a controlled study, retreat-based spa rehabilitation programs should be introduced as an inseparable element of the comprehensive treatment offered to people at a chronic stage of recovery after ischemic strokes. The findings suggest that the short-term effects are not related to the gender, age or time from stroke onset, while the follow-up exam showed that the long-term effects may depend on the time from stroke onset. Furthermore, the quality of life related to physical and mental health in patients 1–5 years from stroke onset is likely to decrease faster. Nevertheless, at the follow-up exam, the related scores were still higher than before the therapy program. As for the patients’ physical health, the effects do not seem to be related to sex. Patients below 65 years of age and patients 1–5 years from stroke onset presented higher functional efficiency at the end of the spa rehabilitation program (short-term effect) and at the follow-up (long-term effect). In view of the fact that stroke patients require life-long therapy, and their neural plasticity may be enhanced by various types of exercise, it is likely that spa rehabilitation programs, applied as a complementary therapy, would be beneficial not only at the chronic stage but also at an early stage of recovery post-stroke. To confirm this hypothesis, it is necessary to continue the related research in controlled studies involving larger cohorts and taking into account patients at earlier stages of recovery from strokes.

## Figures and Tables

**Table 1:** Characteristics of each patient’s baseline status.

Variable		Mean/N	SD
Age		66.31	8.20
Sex	Female	16	50.0%
	Male	16	50.0%
Education	Primary	3	9.4%
	Secondary	8	25.0%
	Vocational	10	31.3%
	Higher	11	34.4%
Residency	With spouse and children	18	56.3%
	With spouse	11	34.4%
	With children	3	9.4%
	Alone	0	0.00%
Place of residence	Urban area	19	59.4%
	Rural area	13	40.6%
Type of stroke	Ischemic	32	100.0%
Time from stroke	1–5 years	13	40.6%
	over 5–10 years	19	59.4%
Rankin	2: Slight disability	3	9.4%
	3: Moderate disability	29	90.6%
FAC	3: Ambulator, dependent on supervision	29	90.6%
	4: Ambulator, independent, level surfaces only	3	9.4%
Barthel	Feeding	6.41	2.28
	Transfers (bed to chair and back)	7.19	2.52
	Personal hygiene	6.41	2.28
	Toilet use	7.34	2.54
	Bathing, washing the whole body	5.47	1.48
	Mobility on level surfaces	6.09	2.10
	Stair climbing	5.31	1.23
	Dressing and undressing	5.47	1.48
	Bladder control	9.84	0.88
	Bowel control	9.06	1.98
Total Barthel Index before		68.59	9.61

SD: standard deviation, N: number of subjects; %: percent of subjects, FAC: Functional Ambulation Category.

**Table 2:** WHOQOL-BREF and Barthel scores in Exams I, II and III for the whole group.

	Exam I (*n* = 32)		Exam II (*n* = 32)		Exam III (*n* = 31)		*p* (II vs. I)	*p* (III vs. I)	*p* (III vs. II)
	Mean	SD	Mean	SD	Mean	SD			
WHOQOL-BREF									
Individual overall perception of quality of life	2.88	0.66	3.19	0.64	2.96	0.84	0.001	0.415	0.011
Individual overall perception of quality of health	2.56	0.80	3.19	0.64	3.00	0.77	0.000	0.001	0.022
Somatic	47.43	7.16	57.37	7.53	49.74	6.29	0.000	0.035	0.000
Psychological	46.22	14.68	55.73	11.29	49.85	11.08	0.000	0.017	0.000
Social	56.51	15.22	63.54	14.93	61.31	14.02	0.000	0.048	0.005
Environmental	55.08	14.22	62.01	13.80	58.37	14.18	0.000	0.000	0.000
Barthel Index									
Feeding	6.41	2.28	7.97	2.49	7.86	2.52	0.001	0.006	1.000
Transfers (bed to chair and back)	7.19	2.52	8.28	2.41	8.04	2.49	0.006	0.083	0.161
Personal hygiene	6.41	2.28	7.81	2.52	7.50	2.55	0.002	0.022	0.326
Toilet use	7.34	2.54	8.75	2.20	8.57	2.30	0.002	0.011	1.000
Bathing, washing the whole body	5.47	1.48	5.63	1.68	5.54	1.57	0.325	1.000	1.000
Mobility on level surfaces	6.09	2.10	8.59	2.28	8.39	2.38	0.000	0.000	0.161
Stair climbing	5.31	1.23	7.50	2.54	6.96	2.49	0.000	0.001	0.083
Dressing and undressing	5.47	1.48	6.25	2.20	6.07	2.09	0.023	0.043	1.000
Bladder control	9.84	0.88	9.84	0.88	9.82	0.94	1.000	1.000	1.000
Bowel control	9.06	1.98	9.22	1.84	9.29	1.78	0.325	1.000	1.000
Total Barthel Index	68.59	9.61	79.84	11.32	77.68	11.98	0.000	0.000	0.039

**Table 3:** The effects of the rehabilitation program relative to the age, sex and time from stroke onset of patients.

		Sex				*p*	Age				*p*	Time from Stroke				*p*
		Female		Male			<65 years		65 Years and Over			1–5 Years		>5–10 Years		
		Mean	SD	Mean	SD		Mean	SD	Mean	SD		Mean	SD	Mean	SD	
WHOQOL-BREF																
Effect I	Somatic	9.38	4.36	10.50	6.03	0.550	11.46	5.72	8.89	4.70	0.175	12.00	5.60	8.53	4.55	0.063
	Psychological	9.13	7.46	10.00	6.45	0.725	9.38	7.19	9.68	6.84	0.906	11.38	5.91	8.32	7.35	0.220
	Social	8.25	6.21	5.69	6.68	0.270	5.54	3.84	7.95	7.74	0.255	4.92	4.05	8.37	7.50	0.104
	Environmental	6.38	4.50	7.31	4.64	0.566	7.62	4.52	6.32	4.57	0.434	7.85	4.51	6.16	4.52	0.307
Effect II	Somatic	0.50	5.02	3.93	5.20	0.087	2.42	6.54	2.06	4.39	0.873	2.42	6.54	2.06	4.39	0.873
	Psychological	1.14	7.40	6.29	7.54	0.080	2.50	6.82	4.63	8.54	0.485	3.17	6.73	4.13	8.69	0.754
	Social	4.79	7.86	1.21	7.08	0.218	2.00	4.97	3.75	9.13	0.523	0.67	5.35	4.75	8.62	0.136
	Environmental	2.79	3.98	3.00	3.53	0.881	4.25	3.49	1.88	3.61	0.093	3.75	3.65	2.25	3.71	0.296
Effect III	Somatic	−8.86	4.28	−6.21	3.98	0.103	−9.08	3.90	−6.38	4.29	0.098	−9.67	4.08	−5.94	3.79	0.019
	Psychological	−7.43	5.87	−4.00	3.14	0.068	−7.25	5.82	−4.56	3.97	0.158	−8.58	4.74	−3.56	3.98	0.005
	Social	−3.50	7.05	−3.50	5.36	1.000	−3.33	4.12	−3.63	7.45	0.904	−4.08	5.60	−3.06	6.67	0.672
	Environmental	−3.00	2.35	−3.86	3.42	0.446	−3.00	2.22	−3.75	3.38	0.485	−3.75	2.60	−3.19	3.19	0.622
Barthel Index																
Effect I	Total Barthel Index	10.94	6.64	11.56	8.31	0.816	12.69	7.53	10.26	7.35	0.371	12.31	8.07	10.53	7.05	0.513
Effect II	Total Barthel Index	9.29	6.16	6.79	6.68	0.313	11.67	6.85	5.31	4.64	0.007	11.25	7.42	5.63	4.43	0.032
Effect III	Total Barthel Index	−0.36	1.34	−3.21	5.75	0.091	0.00	0.00	−3.13	5.44	0.036	0.00	0.00	−3.13	5.44	0.036

## Data Availability

Not applicable.

## Supplementary Materials

The following are available online at https://www.mdpi.com/article/10.3390/brainsci11040501/s1. Table S1. The effects of the rehabilitation program relative to age, sex and time from stroke onset.
