# Supplementary material for: The Efficiency of Spa Rehabilitation in Chronic Ischemic Stroke Patients—Preliminary Reports
Source: Brain Sci. 2021 Apr 15;11(4):501. doi: 10.3390/brainsci11040501 (PMC8071377; doi:10.3390/brainsci11040501)
Supplement: Supplementary file 1 [file brainsci-11-00501-s001.zip › brainsci-1170483-supplementary.pdf]

Table S1. The effects of the rehabilitation program relative to age, sex and time from stroke onset

		Sex				p	Age				p	Time from stroke				p
		Female		Male			<65 years		65 years and over			1-5 years		>5-10 years		
		Mean	SD	Mean	SD		Mean	SD	Mean	SD		Mean	SD	Mean	SD	
**WHOQOL-Bref**																
**Effect I**	**Individual general perception of quality of life**	0.31	0.48	0.31	0.48	1.000	0.46	0.52	0.21	0.42	0.161	0.46	0.52	0.21	0.42	0.161
	**Individual general perception of quality of health**	0.56	0.51	0.69	0.60	0.532	0.77	0.44	0.53	0.61	0.201	0.85	0.38	0.47	0.61	**0.041**
	**Somatic**	9.38	4.36	10.50	6.03	0.550	11.46	5.72	8.89	4.70	0.175	12.00	5.60	8.53	4.55	0.063
	**Psychological**	9.13	7.46	10.00	6.45	0.725	9.38	7.19	9.68	6.84	0.906	11.38	5.91	8.32	7.35	0.220
	**Social**	8.25	6.21	5.69	6.68	0.270	5.54	3.84	7.95	7.74	0.255	4.92	4.05	8.37	7.50	0.104
	**Environmental**	6.38	4.50	7.31	4.64	0.566	7.62	4.52	6.32	4.57	0.434	7.85	4.51	6.16	4.52	0.307
**Effect II**	**Individual general perception of quality of life**	0.07	0.83	0.14	0.53	0.789	0.25	0.62	0.00	0.73	0.349	0.33	0.49	-0.06	0.77	0.133
	**Individual general perception of quality of health**	0.43	0.76	0.57	0.65	0.596	0.58	0.67	0.44	0.73	0.592	0.67	0.49	0.38	0.81	0.279
	**Somatic**	0.50	5.02	3.93	5.20	0.087	2.42	6.54	2.06	4.39	0.873	2.42	6.54	2.06	4.39	0.873
	**Psychological**	1.14	7.40	6.29	7.54	0.080	2.50	6.82	4.63	8.54	0.485	3.17	6.73	4.13	8.69	0.754
	**Social**	4.79	7.86	1.21	7.08	0.218	2.00	4.97	3.75	9.13	0.523	0.67	5.35	4.75	8.62	0.136
	**Environmental**	2.79	3.98	3.00	3.53	0.881	4.25	3.49	1.88	3.61	0.093	3.75	3.65	2.25	3.71	0.296
**Effect III**	**Individual general perception of quality of life**	-0.29	0.47	-0.14	0.36	0.376	-0.17	0.39	-0.25	0.45	0.611	-0.08	0.29	-0.31	0.48	0.129
	**Individual general perception of quality of health**	-0.14	0.36	-0.21	0.43	0.637	-0.25	0.45	-0.13	0.34	0.412	-0.25	0.45	-0.13	0.34	0.412
	**Somatic**	-8.86	4.28	-6.21	3.98	0.103	-9.08	3.90	-6.38	4.29	0.098	-9.67	4.08	-5.94	3.79	**0.019**
	**Psychological**	-7.43	5.87	-4.00	3.14	0.068	-7.25	5.82	-4.56	3.97	0.158	-8.58	4.74	-3.56	3.98	**0.005**
	**Social**	-3.50	7.05	-3.50	5.36	1.000	-3.33	4.12	-3.63	7.45	0.904	-4.08	5.60	-3.06	6.67	0.672
	**Environmental**	-3.00	2.35	-3.86	3.42	0.446	-3.00	2.22	-3.75	3.38	0.485	-3.75	2.60	-3.19	3.19	0.622
**Index Barthel**																
**Effect I**	**Eating meals**	1.88	2.50	1.25	2.24	0.462	1.92	2.53	1.32	2.26	0.483	1.92	2.53	1.32	2.26	0.483
	**Moving from bed to chair and back**	0.94	2.02	1.25	2.24	0.681	1.54	2.40	0.79	1.87	0.330	1.54	2.40	0.79	1.87	0.330
	**Maintaining personal hygiene**	1.25	2.24	1.56	2.39	0.705	1.92	2.53	1.05	2.09	0.297	1.92	2.53	1.05	2.09	0.297
	**Using the toilet**	1.56	2.39	1.25	2.24	0.705	1.15	2.19	1.58	2.39	0.613	0.77	1.88	1.84	2.48	0.174
	**Bathing, washing the whole body**	0.31	1.25	0.00	0.00	0.333	0.00	0.00	0.26	1.15	0.417	0.00	0.00	0.26	1.15	0.417
	**Moving on flat surfaces**	2.50	2.58	2.50	2.58	1.000	3.08	2.53	2.11	2.54	0.295	3.08	2.53	2.11	2.54	0.295
	**Going up and down stairs**	2.19	2.56	2.19	2.56	1.000	2.69	2.59	1.84	2.48	0.357	2.31	2.59	2.11	2.54	0.828
	**Dressing and undressing**	0.31	1.25	1.25	2.24	0.156	0.38	1.39	1.05	2.09	0.286	0.77	1.88	0.79	1.87	0.976
	**Controlling urine excretion**	0.00	0.00	0.00	1.83	1.000	0.00	0.00	0.00	1.67	1.000	0.00	0.00	0.00	1.67	1.000
	**Controlling stool excretion**	0.00	0.00	0.31	1.25	0.333	0.00	0.00	0.26	1.15	0.417	0.00	0.00	0.26	1.15	0.417
	**Total Index Barthel**	10.94	6.64	11.56	8.31	0.816	12.69	7.53	10.26	7.35	0.371	12.31	8.07	10.53	7.05	0.513
**Effect II**	**Eating meals**	1.43	2.34	1.07	2.13	0.676	1.67	2.46	0.94	2.02	0.397	1.67	2.46	0.94	2.02	0.397
	**Moving from bed to chair and back**	0.71	1.82	0.36	1.34	0.558	1.25	2.26	0.00	0.00	0.082	1.25	2.26	0.00	0.00	0.082
	**Maintaining personal hygiene**	1.07	2.13	0.71	1.82	0.637	1.67	2.46	0.31	1.25	0.101	1.67	2.46	0.31	1.25	0.101
	**Using the toilet**	1.07	2.13	1.07	2.13	1.000	1.25	2.26	0.94	2.02	0.703	0.83	1.95	1.25	2.24	0.611

	**Bathing, washing the whole body**	0.00	0.00	0.00	0.00		0.00	000	0.00	0.00		0.00	0.00	0.00	0.00	
	**Moving on flat surfaces**	2.86	2.57	1.79	2.49	0.272	2.92	2.57	1.88	2.50	0.291	2.92	2.57	1.88	2.50	0.291
	**Going up and down stairs**	1.79	2.49	1.43	2.34	0.699	2.50	2.61	0.94	2.02	0.100	2.08	2.57	1.25	2.24	0.369
	**Dressing and undressing**	0.36	1.34	1.07	2.13	0.299	0.42	1.44	0.94	2.02	0.454	0.83	1.95	0.63	1.71	0.766
	**Controlling urine excretion**	0.00	0.00	0.00	1.96	1.000	0.00	0.00	0.00	1.83	1.000	0.00	0.00	0.00	1.83	1.000
	**Controlling stool excretion**	0.00	0.00	0.00	0.00		0.00	0.00	0.00	0.00		0.00	0.00	0.00	0.00	
	**Total Index Barthel**	9.29	6.16	6.79	6.68	0.313	11.67	6.85	5.31	4.64	**0.007**	11.25	7.42	5.63	4.43	**0.032**
**Effect III**	**Eating meals**	0.00	0.00	0.00	0.00		0.00	0.00	0.00	0.00		0.00	0.00	0.00	0.00	
	**Moving from bed to chair and back**	0.00	0.00	-0.71	1.82	0.165	0.00	0.00	-0.63	1.71	0.164	0.00	0.00	-0.63	1.71	0.164
	**Maintaining personal hygiene**	0.00	0.00	-0.36	1.34	0.336	0.00	0.00	-0.31	1.25	0.397	0.00	0.00	-0.31	1.25	0.397
	**Using the toilet**	0.00	0.00	0.00	0.00		0.00	0.00	0.00	0.00		0.00	0.00	0.00	0.00	
	**Bathing, washing the whole body**	0.00	0.00	0.00	0.00		0.00	0.00	0.00	0.00		0.00	0.00	0.00	0.00	
	**Moving on flat surfaces**	0.00	0.00	-0.71	1.82	0.165	0.00	0.00	-0.63	1.71	0.164	0.00	0.00	-0.63	1.71	0.164
	**Going up and down stairs**	-0.36	1.34	-0.71	1.82	0.558	0.00	0.00	-0.94	2.02	0.083	0.00	0.00	-0.94	2.02	0.083
	**Dressing and undressing**	0.00	0.00	0.00	0.00		0.00	0.00	0.00	0.00		0.00	0.00	0.00	0.00	
	**Controlling urine excretion**	0.00	0.00	0.00	0.00		0.00	0.00	0.00	0.00		0.00	0.00	0.00	0.00	
	**Controlling stool excretion**	0.00	0.00	0.00	0.00		0.00	0.00	0.00	0.00		0.00	0.00	0.00	0.00	
	**Total Index Barthel**	-0.36	1.34	-3.21	5.75	0.091	0.00	0.00	-3.13	5.44	**0.036**	0.00	0.00	-3.13	5.44	**0.036**

*independent samples t-test
